# Screening for Psychological Distress in Vietnamese Cancer Patients: An Evaluation of the Distress Thermometer

**DOI:** 10.1002/cam4.4298

**Published:** 2021-09-24

**Authors:** Tien Quang Nguyen, Tuyet Mai Do, Tuan Anh Pham

**Affiliations:** ^1^ Vietnam National Cancer Hospital Hanoi Vietnam

**Keywords:** cancer, distress thermometer, psychological distress, screening

## Abstract

**Background:**

Psychological distress is prevalent in patients with cancer, negatively affecting their treatment and quality of life. Clinical guidelines recommended screening all cancer patients routinely for psychological problems using simple measures such as the Distress Thermometer (DT) and Problem List (PL). This study is the first research in Vietnam to identify the optimal DT cutoff point to screen distress and the relationship with PL items among cancer patients.

**Methods:**

300 cancer patients were recruited from 10 departments at Vietnam National Cancer Hospital (K hospital) and completed the DT and PL. Participants also completed the Patient Health Questionnaire‐9 (PHQ‐9) and the Generalized Anxiety Disorder‐7 (GAD‐7) with standard cutoff scores for identifying significant depression and anxiety.

**Results:**

Receiver operating characteristic (ROC) curve analyses showed that a DT cutoff score of 4 had an area under the ROC curve of 0.81 and 0.82 using the PHQ‐9 and GAD‐7 cutoff scores of 10 as the criterion, respectively. This indicated good overall accuracy. This cutoff also showed a sensitivity of 0.87 and 0.92 for PHQ‐9 and GAD‐7 total score defined cases, respectively. Both specificity values were 0.58. In terms of the PL, 164 distressed patients (54.7%) reported significantly more emotional problems, family issues, and practical and physical problem, implying various causes contribute to psychological distress among cancer patients.

**Conclusions:**

The study showed that the DT with a cutoff of 4 accompanied with PL is a simple and effective instrument compared to previous, longer measures commonly used to detect psychosocial distress in Vietnamese cancer patients. This cutoff point also identified patients with problems contributing towards distress.

## Graphical abstract

The psychosocial aspects of cancer are usually underestimated and unrecognized. It is of necessity to use a short and simple screening measure for significant psychological problems of this vulnerable population. Our study is the first study in Vietnam to prove that the Distress Thermometer with the cutoff score of 4 accompanied with the Problem List is valid to Vietnamese people with cancer.

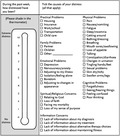

## 1 INTRODUCTION

According to the Global Cancer Observatory 2020 (GCO), Vietnam witnessed 182,563 new cases of cancer and 122,690 deaths due to cancer.^1^ Surveys found that 20% to 52% of cancer patients had a remarkable level of distress related to cancer diagnosis and treatment, especially in difficult times of life ^2^, ^3^, ^4^. It is a fact that distress is common in the cancer population, especially depression and anxiety, but unrecognized by oncology professionals, especially depression and anxiety ^5^, ^6^. These underestimated psychological problems can cause many negative outcomes, such as poor adherence to treatment and reduced satisfaction with care, leading to decreased effectiveness of treatment and lower quality of life ^7^, ^8^, ^9^, ^10^. For this reason, psychosocial care should be considered an important part of standard treatment for cancer. Consequently, distress screening should be integrated into routine care ^11^, ^12^.

According to the National Comprehensive Cancer Network (NCCN), clinicians should screen routinely for psychological distress in all cancer patients, at all stages and settings.^13^ In the context of the clinical practice of Vietnam, the 9‐item Patient Health Questionnaire (PHQ‐9) and 7‐item Generalized Anxiety Disorder (GAD‐7) have been commonly used to screen for depression and anxiety in cancer patients. However, it takes time and effort to score and interpret these multi‐item screeners. Moreover, these measures do not have a wide enough spectrum to identify the diverse psychological problems of cancer patients.

To meet the need of rapid screening of distress in cancer patients, the single‐item Distress Thermometer (DT) was developed by Roth and colleagues for patients to rate distress from 0 (“no distress”) to 10 (“extreme distress”). The DT cutoff point has changed slightly in different research ^14^, ^15^, ^16^, ^17^. Holland identified 4 as the best cutoff to identify clinical distress in a cancer patient,^14^ and the NCCN Clinical Practice Guidelines for Distress Management recommends using a DT cutoff score of 4 followed by a Problem List (PL) to investigate any unmet psychological needs in the prior week. Firstly, the cancer patients circle the number indicating the level of distress on a 0 to 10 scale (DT), then are asked to complete the PL, including problems from 5 areas of life. In other words, the PL helps to identify the cause of patients’ distress so that the patients will be correctly referred in subsequent steps of care.^18^

The research investigated the efficacy of the DT to screen psychological distress against other longer measures. Previously, the DT has been proved to be valid in other Asian countries such as Korea, Japan, and Taiwan with an optimal cutoff ≥4 and sensitivity >80% ^19^, ^20^, ^21^. Setting a cutoff of 5 in DT and using the established cutoff of 10 in PHQ‐9 and GAD‐7, Baba found that 33.3% of patients had distress; 16.5% and 13% of participants reported depression and anxiety, respectively. The concordance rate between the 3 screening tests was not reported.^22^ In another study, Hegel demonstrated that ROC analyses showed that DT had stronger power compared to the PHQ‐9 for depression in cancer patients. The DT cutoff of 4 had a sensitivity of 100% and specificity of 45% for depression screening.^23^ In terms of anxiety, no further information regarding any differences in screening performance between the DT and GAD‐7 was identified.

In Vietnam, the mental health of cancer patients is receiving more and more attention from clinicians and policymakers. In fact, the PHQ‐9 and GAD‐7 are used frequently to screen for psychological problems in Vietnamese patients, but in many cases, oncology clinicians only have enough time to perform a simple test such as the DT. To date, there has been no study assessing the application of the DT to screen for distress in cancer patients in Vietnam. Therefore, we conducted this study to examine the operating characteristics of the DT as a screening measure for distress among the cancer population in Vietnam. The aims of this study were to (1) to find the optimal cutoff score on the DT to screen for distress and (2) to investigate the relationship between the DT cutoff score and PL items.

## 2 MATERIALS AND METHODS

### 2.1 Study design

A cross‐sectional study.

### 2.2 Sample

The data was collected between July 2020 and September 2020. Using the sample size calculation formula in cross‐sectional studies described by Mohamad^24^ with expected prevalence of 0.2, desired precision of 0.05 and confidence level of 0.95, the required sample size was 246. In this study, 300 cancer patients at 10 departments of Vietnam National Cancer Hospital in Hanoi participated in the study. These departments included 4 internal oncology departments, 2 surgical oncology departments, 2 radiation oncology departments, 1 multimodal treatment department, and 1 palliative care department. The eligibility requirements consisted of (1) age ≥18 years; (2) having confirmed cancer; (3) being able to understand Vietnamese; and (4) being able to provide consent forms. Those who had severe physical or cognitive problems were excluded from the study.

### 2.3 Procedure

Participants were interviewed in the departments’ treatment rooms. After receiving information about the study and providing their consent form, all participants completed a set of questionnaires including demographic and clinical information, the Distress Thermometer (DT) accompanied with the Problem List (PL), the Patients Health Questionnaire‐9 (PHQ‐9) and the Generalized Anxiety Disorder‐7 (GAD‐7). Of the 300 individuals who were approached, 100% agreed to participate in this study.

### 2.4 Measures

Demographic data were obtained with a questionnaire including age, gender, marital status, educational level, profession and insurance status. Data about disease and treatment was collected via medical records with variables including tumor stage (according to UICC TNM), treatment method and months from diagnosis.

**FIGURE 1:**
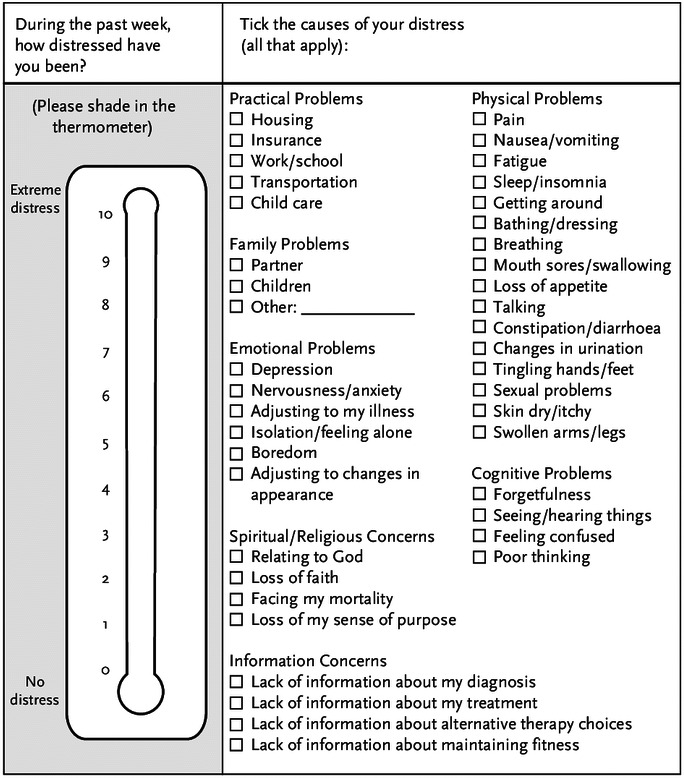
The Distress Thermometer and Problem List (NCCN screening tools for measuring distress)

The DT is a one‐item, self‐report instrument that measures distress by a vertical visual scale ranging from the lowest point (0) (no distress) to the highest point (10) (extreme distress). Patients are guided to circle a number that describes their most appropriate level of distress in the last week (Figure 1). The Vietnamese version of the DT and PL was forward‐translated (from English to Vietnamese) by a clinical professional who has knowledge of English‐speaking culture, and was then checked by a bilingual expert and back‐translated (from Vietnamese to English) by an independent translator and pre‐tested carefully before use on the target population. The characteristics of the DT with a cutoff score of 4 are the subject of this study.

The Problem List (PL) was developed by the Distress Management Guidelines Panel of the NCCN to identify the common causes of distress experienced by those with cancer. These contributing factors are grouped into 7 categories (practical problems, family problems, emotional problems, spiritual/religious concerns, physical problems, information concerns). Patients completed the list by choosing yes or no for each item listed that could have caused distress within the previous week (Figure 1).

The PHQ‐9 is a nine‐item, self‐report commonly used for screening depression in the Vietnamese population. This instrument measures the presence and severity of depressive symptoms according to the criteria of the DSM‐IV.^25^ The participant is asked to rate how each symptom bothered them within the previous 2 weeks. Each item is scored on a Likert‐type scale as follows: 0 (not at all); 1(several days); 2 (more than half the time); and 3 (nearly every day). A score of 0–4 suggests no depression; scores of 5–9 shows mild depression; a score of 10–14 represent moderate depression; and a score ≥15 indicates severe depression. This study used the PHQ‐9 cutoff point ≥10 with specificity and sensitivity of 88% for major depression.^9^, ^26^

The GAD‐7 is a seven‐item questionnaire to assess the 7 main symptoms of anxiety disorder. Patients rate the frequency of each symptom on a four‐point scale ranging from 0 (not at all) to 3 (almost every day) within 2 weeks. A score of ≤4 shows no anxiety, a score of 5–9 illustrates mild anxiety, a score of 10–14 reveals moderate anxiety, and a score ≥15 represents severe anxiety. A cut‐off of 10 has been identified as the optimal point for sensitivity (89%) and specificity (82%).^27^

The Vietnamese versions of PHQ‐9 and GAD‐7 have been translated and used most widely in clinical settings. In this study, we used 2 measures (PHQ‐9 and GAD‐7) with cutoff scores of 10 as gold standards for identifying psychological distress (depression and anxiety) in cancer patients.

### 2.5 Statistical analysis

The questionnaire forms were coded according to the time of the interview. Data were entered, then analyzed using SPSS version 20.0. Univariate analysis was used to identify frequency and proportion. Receiver Operating Characteristics (ROC) curve analyses were used to visualize the sensitivity and 1‐specificity for each score on the DT compared to the PHQ‐9 and GAD‐7 cutoffs. The Area Under the Curve (AUC) of each ROC curve represents the DT’s accuracy relative to the cutoff points on the PHQ‐9 and GAD‐7. An AUC of 1 shows perfect accuracy, while an AUC of 0.5 suggests no significant accuracy compared to the established criteria. Chi‐squared analyses were used to investigate the relationship between the DT cutoff and the notification of reported items on the PL. Fisher's exact test was used when more than 20% of cells had expected frequencies <5. All tests were two‐tailed using a significance level of α < 0.05.

The study was approved by the Research Ethics Committee of the Vietnam National Cancer Hospital.

## 3 RESULTS

### 3.1 Demographic and clinical characteristics

Table 1 illustrates that 300 patients in the study had an average age of 54 years (ranging from 18 to 80). The sample was divided relatively evenly by gender (51% female and 49% male). Most participants were married (89%), literate (96.7%), and working (82%). Among a wide range of observed cancer diagnoses of the sample, the three most common types were colorectal cancer (21.7%), breast cancer (20.3%), and stomach/esophageal cancer (19.7%). More than sixty percent of the patients had cancer at a late stage (III‐IV). Nearly fifty percent of the participants received chemotherapy and approximately 20% of the sample received surgery and radiation. Participants had been diagnosed with cancer on average 13.44 months previously (ranging from 1 week to 16 years).

**TABLE 1:** Demographic and clinical characteristics of cancer patients

Variable	No. of patients	%	
Age in years (Mean ±SD)	54.9 ± 11.5		
Gender			
Women	153	51.0	
Men	147	49.0	
Marital status			
Single	10	3.3	
Married	267	89.0	
Divorced	5	1.7	
Widow	18	6.0	
Education			
Illiterate	10	3.3	
Primary school	20	6.7	
Secondary school	136	45.3	
High school	90	30.0	
College or higher	44	14.7	
Working status		
Yes	244	82.0	
No	54	18.0	
Cancer diagnosis			
Colon/rectum (C17‐21)	65	21.7	
Breast (C50)	61	20.3	
Stomach/esophagus (C15‐16)	59	19.7	
Lung (C34)	34	11.3	
Female genital organs (C51‐57)	27	9.0	
Head and neck (C02‐15, C32)	22	7.3	
Hepatobiliary (C22‐23)	7	2.3	
Thyroid (C73)	2	0.7	
Pancreas (C25)	2	0.7	
Other	21	7.0	
Tumor stage (UICC TNM)			
I	28	9.3	
II	81	27.0	
III	130	43.3	
IV	61	20.3	
Current treatment method			
Surgery	61	20.3	
Radiation	58	19.3	
Chemotherapy	147	49.0	
Other	34	11.4	
Months from diagnosis (Mean ±SD)	13.44 ± 26.88		

### 3.2 Establishment of a DT cutoff score

**FIGURE 2:**
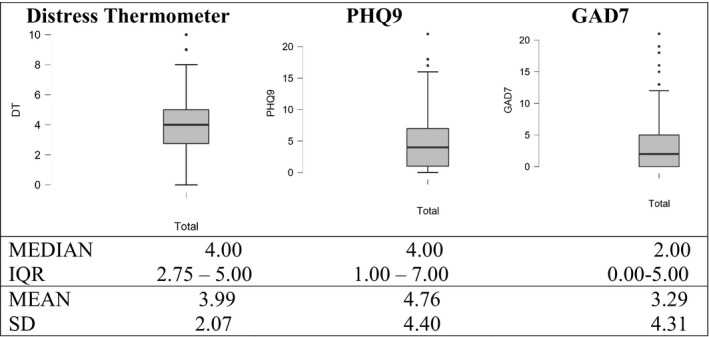
Box plots of data from Distress Thermometer, PHQ‐9 and GAD‐7 tests. The boundary of the box closest to zero indicates the 25th percentile, a black line within the box marks the median, and the boundary of the box farthest from zero indicates the 75th percentile. Whiskers above and below the box indicate the 10th and 90th percentiles. Points above and below the whiskers indicate outliers outside the 10th and 90th percentiles. Text above each box plot indicates the corresponding tests. Median, IQR (interquantile range), mean, and SD (standard deviation) are presented at the bottom outside the graph

**FIGURE 3:**
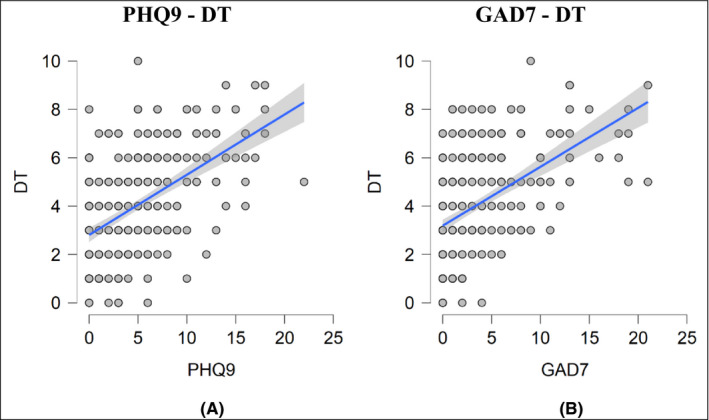
Scatter plots of correlation between DT and other tests. (A) The relationship between the PHQ‐9 and DT scoring. A linear regression analysis yielded the line in blue with *r* = 0.531 (95% CI of 0.444 to 0.607, *p* value <0.001). (B) The relationship between the GAD‐7 – DT scoring. A linear regression analysis yielded the line in blue with *r* = 0.509 (95% CI of 0.420 to 0.588, *p* value <0.001)

**FIGURE 4:**
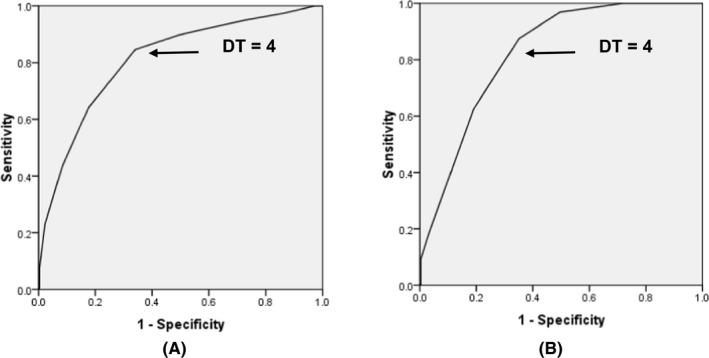
Receiver operating characteristic curve analysis comparing Distress Thermometer (DT) scores with (A) established Patient Health Questionnaire 9 (PHQ‐9) cutoff score; (B) established Generalized Anxiety Disorder 7 (GAD‐7) cutoff score

Table 2 showed that 54.7% of cancer patients reported a DT score of 4 or above. The Figure 2 illustrates that the mean score (SD) of the DT was 3.99 (2.066); meanwhile, the mean (SD) of PHQ‐9 score was 4.76 (4.402) and of GAD‐7 was 3.29 (4.314). Figure 3 showed that there was a positive relationship between the PHQ‐9 and DT scoring with r coefficient of 0.531. Similarly, the GAD‐7 and DT scoring had a moderate positive correlation with r coefficient of 0.509. Figure 4 represents the ROC curves with the sensitivity and 1‐specificity for every point on the DT compared to the PHQ‐9 (a) and GAD‐7 (b) cutoff points to identify distress in the study sample. Based on previous studies ^9^, ^26^, ^27^ distress, specifically depression and anxiety, was identified as a total score ≥10 on the PHQ‐9 or GAD‐7. In this study, the AUC was 0.81 and 0.82 compared to the PHQ‐9 and GAD‐7 cutoff scores of 10, respectively. These values represent the good overall accuracy of this test, suggesting a score ≥4 is the optimal DT cutoff score for screening distress in the Vietnamese cancer population. Setting PHQ‐9 as the criterion, it was found that a DT cutoff score of 4 had a sensitivity of 0.87 and a specificity of 0.58 for detecting depression. Similarly, compared to the GAD‐7, the DT cutoff of 4 showed a sensitivity of 0.92 and a specificity of 0.58 for identifying anxiety.

**TABLE 2:** Frequency distribution of Distress Thermometer scores

Score	No. of patients	%	Cumulative %
0	7	2.3	2.3
1	30	10.0	12.3
2	38	12.7	25.0
3	61	20.3	45.3
4	42	14.0	59,3
5	51	17.0	76.3
6	32	10.7	87.0
7	24	8.0	95.0
8	11	3.7	98.7
9	3	1.0	99.7
10	1	0.3	100

### 3.3 Relationship between the DT cutoff and problem list

Table 3 investigates the relationship between the DT cutoff of 4 and the reported items of the PL.

**TABLE 3:** Relation of the DT cutoff score to Problem List items

Variable		DT: no. of patients (%)			OR (95%CI)	p‐value
		< 4	≥ 4			
Practical problems						
Housing	Yes	9 (6.6)	27 (16.5)		0.36 (0.16–0.79)	0.009
	No	127 (93.4)	137 (83.5)			
Insurance	Yes	7 (5.1)	20 (12.2)		0.39 (0.16–0.95)	0.034
	No	129 (94.0)	144 (87.8)			
Work/school	Yes	12 (8.8)	31 (18.9)		0.42 (0.20–0.84)	0.013
	No	124 (91.2)	133 (81.1)			
Transportation	Yes	4 (2.9)	7 (4.3)		0.68^a^ (0.19–2.37)	0.759
	No	132 (97.1)	151 (95.1)			
Childcare	Yes	16 (11.8)	27 (16.5)		0.677 (0.35–1.32)	0.248
	No	120 (88.2)	137 (83.5)			
Family problems						
Partner	Yes	0 (0)	7 (4.3)		^a^	0.017
	No	136 (100)	157 (95.7)			
Children	Yes	1 (0.7)	22 (13.4)		0.05 (0.01–0.36)	< 0.001
	No	135 (99.3)	142 (86.6)			
Emotional problems						
Depression	Yes	21 (15.4)	103 (62.8)		0.11 (0.06–0.19)	< 0.001
	No	115 (84.6)	61 (37.2)			
Anxiety/nervousness	Yes	44 (32.2)	116 (70.7)		0.20 (0.12–0.32)	< 0.001
	No	92 (67.6)	48 (29.3)			
Adjusting to illness	Yes	9 (6.6)	31 (18.9)		0.30 (0.14–0.66)	0.002
	No	127 (93.4)	133 (81.1)			
Isolation	Yes	3 (2.2)	22 (13.4)		0.14 (0.04–0.50)	< 0.001
	No	133 (97.8)	142 (86.6)			
Boredom	Yes	6 (4.4)	54 (32.9)		0.09 (0.04–0.23)	< 0.001
	No	130 (95.6)	110 (67.1)			
Adjusting to changes	Yes	12 (8.8)	11 (6.7)		1.35 (0.57–3.16)	0.493
	No	124 (91.2)	153 (93.6)			
Spiritual/Religious concerns						
Relating to God	Yes	25 (18.4)	22 (13.4)		1.45 (0.80–2.71)	0.239
	No	111 (81.6)	142 (86.6)			
Loss of faith	Yes	2 (1.5)	6 (3.7)		0.39^a^ (0.08–1.98)	0.3
	No	134 (98.5)	158 (96.3)			
Facing mortality	Yes	1 (0.7)	6 (3.7)		0.20^a^ (0.02–1.64)	0.132
	No	135 (99.3)	158 (96.3)			
Loss of purpose	Yes	0 (0)	5 (3.0)		^a^	0.066
	No	136 (100)	159 (97.0)			
Physical problems						
Pain	Yes	38 (27.9)	88 (53.7)		2.99 (1.84–4.85)	< 0.001
	No	98 (72.1)	76 (46.3)			
Nausea/vomiting	Yes	26 (19.1)	43 (26.2)		1.50 (0.87–2.61)	0.146
	No	110 (80.9)	121 (73.8)			
Fatigue	Yes	45 (33.1)	108 (65.9)		3.90 (2.41–6.31)	< 0.001
	No	91 (66.9)	56 (34.1)			
Sleep/insomnia	Yes	65 (47.8)	112 (68.3)		2.35 (1.47–3.77)	< 0.001
	No	71 (52.2)	52 (31.7)			
Getting around	Yes	15 (11.0)	29 (17.7)		1.73 (0.89–3.39)	0.105
	No	121 (89.0)	135 (82.3)			
Bathing/dressing	Yes	8 (5.9)	16 (9.8)		1.73 (0.72–4.18)	0.218
	No	128 (94.1)	148 (90.2)			
Breathing	Yes	5 (3.7)	19 (11.6)		3.43 (1.25–9.46)	0.012
	No	131 (96.3)	145 (88.4)			
Mouth sores/swallowing	Yes	23 (16.9)	25 (15.2)		0.88 (0.48–1.64)	0.695
	No	113 (83.1)	139 (84.8)			
Loss of appetite	Yes	43 (31.6)	70 (42.7)		1.61 (1.00–2.59)	0.049
	No	93 (68.4)	94 (57.3)			
Talking	Yes	7 (5.1)	8 (4.9)		0.95 (0.33–2.68)	0.915
	No	129 (94.9)	156 (95.1)			
Constipation/ diarrhea	Yes	41 (30.1)	63 (38.4)		1.45 (0.89–2.34)	0.134
	No	95 (69.9)	101 (65.6)			
Changes in urination	Yes	7 (5.1)	12 (7.3)		1.46 (0.56–3.80)	0.442
	No	129 (94.9)	152 (92.7)			
Tingling hands/feet	Yes	48 (35.3)	65 (39.6)		1.20 (0.75–1.93)	0.440
	No	88 (64.7)	99 (60.4)			
Sexual problems	Yes	7 (5.1)	5 (3.0)		0.58 (0.18–1.87)	0.356
	No	129 (94.9)	195 (97.0)			
Skin dry/itchy	Yes	13 (9.6)	19 (11.6)		1.24 (0.59–2.61)	0.571
	No	123 (90.4)	145 (88.4)			
Swollen arms/legs	Yes	4 (2.9)	7 (4.3)		0.25^a^ (0.09–0.72)	0.759
	No	132 (97.1)	157 (95.7)			
Cognitive problems						
Forgetfulness	Yes	36 (26.5)	76 (46.3)		0.42 (0.26–0.68)	< 0.001
	No	100 (73.5)	88 (53.7)			
Seeing/hearing things	Yes	3 (2.2)	4 (2.4)		0.90^a^ (0.20–4.10)	1.0
	No	133 (97.8)	160 (97.6)			
Feeling confused	Yes	15 (11.0)	33 (20.1)		0.49 (0.26–0.95)	0.032
	No	121 (89.0)	131 (79.9)			
Poor thinking	Yes	5 (3.7)	4 (2.4)		1.53^a^ (0.40–5.80)	0.736
	No	131 (96.3)	160 (97.6)			
Information concerns						
Diagnosis	Yes	23 (16.9)	24 (14.6)		1.19 (0.64–2.21)	0.589
	No	113 (83.1)	140 (85.4)			
Treatment	Yes	26 (19.1)	31 (18.9)		1.01 (0.57–1.81)	0.962
	No	110 (80.9)	133 (81.1)			
Alternative choices	Yes	23 (16.9)	27 (16.5)		1.03 (0.56–1.90)	0.917
	No	113 (83.1)	137 (83.5)			
Maintaining fitness	Yes	35 (25.7)	33 (20.1)		1.38 (0.80–2.37)	0.248
	No	101 (74.3)	131 (79.9)			

^a^ Fisher's Exact test with two‐sided test.

Regarding practical problems, the DT cutoff of 4 was related significantly (*p* ≤ 0.05) to 3 of 5 listed problems (60%). Participants who scored ≥4 on the DT were more likely to report difficulty with housing (OR = 0.360), insurance (OR = 0.391), and work/school (OR = 0.415).

As for family issues, the DT cutoff of 4 was associated significantly (*p* ≤ 0.05) with 2 of 2 listed items (100%). Patients who scored ≥4 had more complaints with their children or partner (OR = 0.048).

In terms of emotional problems, the DT cutoff of 4 had a strong relationship (*p* ≤ 0.05) with 5 of 6 listed problems (83%). Patients who had distress were more likely to report symptoms related to depression (OR = 0.108), nervousness (OR = 0.198), boredom (OR = 0.094), isolation (OR = 0.146), and adjusting to illness (OR = 0.304).

Regarding the spiritual domain, the DT cutoff score of 4 was not related significantly (*p* > 0.05) to any of the 4 problems listed (0%).

Concerning physical aspect, the DT cutoff score was significantly related (*p* ≤ 0.05) to 5 of 16 symptoms listed (31%). Distressed patients had more symptoms of pain (OR = 2.986), fatigue (OR = 3.90), insomnia (OR = 2.353), breathing (OR = 3.433), and loss of appetite (OR = 1.611).

In terms of cognitive function, the DT cutoff point of 4 had a significant association (*p* ≤ 0.05) with 1 of 4 possible problems (25%), revealing that participants having scored above 4 were more likely to suffer from forgetfulness (OR = 0.417).

In the information category, the DT cutoff score did not relate significantly (*p* > 0.05) to any of the 4 choices listed (0%).

## 4 DISCUSSION

The main result from this study was that the DT was effective compared to the PHQ‐9 and GAD‐7 as a short measure of screening for psychological problems among Vietnamese cancer patients. This study showed that nearly 55% of cancer patients reported DT scores of 4 and above. This DT cutoff point yielded an optimal sensitivity and specificity against the cutoffs of other instruments. Distressed cancer patients (score ≥4) were significantly more likely to experience several psychosocial problems including practical, family, emotional, cognitive, and physical issues.

It can be concluded that the DT with a cutoff of 4 is similarly effective as a screening measure compared to the PHQ‐9 and GAD‐7, based on the AUC of 0.81 and 0.82, respectively. This conclusion is exactly in line with the NCCN recommendation.^13^ In a similar study, Hegel noticed a similar AUC (0.86) when comparing the DT to PHQ‐9 scores for depression. A previous study showed a score of 4 in the DT was 1.8 times as likely to present in a patient who scored <10 on the PHQ‐9 as opposed to the PHQ‐9 > 10 groups.^23^ It can be concluded that the DT can effectively distinguish between distressed and non‐distress patients using a cutoff point of 4, relative to existing, longer measures in Vietnam.

The results from our study showed that the patients with scores ≥4 on DT were more likely to complain about emotional and family problems (> 80%). The relationship observed between DT scores ≥4 and the reported physical and cognitive problems listed could be explained by the distressing nature of many common cancer symptoms, especially pain, breathing, fatigue, and insomnia.^28^ This finding was consistent with the prior studies demonstrating that most cancer patients identified the physical and emotional problems as distressing ^15^, ^29^. The possible cause for this may be the vulnerability of middle age and late diagnosis (stage III‐IV) of this population. In the practical sphere, it is clearly seen that finance‐related problems had a significant association with the DT cutoff point of 4. This finding might be due to Vietnam being a developing country with considerable economic disparity in its population, and most cancer patients being unable to maintain or obtain jobs. This financial burden arises in almost all cancer patients during the period of treatment and survivorship or death. Our study has not shown any relationship between a DT cutoff score of 4 and spiritual/religious issues or information concerns. Overall, the present study is in agreement with earlier studies in Asia populations. The DT cutoff point of 4 had an association with emotional problems, family issues, practical and physical problems ^15^, ^19^, ^30^. All these factors may contribute to stress in the daily lives of cancer patients, leading to increased psychological distress in general.

### 4.1 Study limitations

Despite the large sample size from ten different clinical departments and the diverse characteristics of the sample, our study still has some limitations. Firstly, there exists a possible selection bias as this was a cross‐sectional study at a single hospital. Secondly, we only used the PHQ‐9 and GAD‐7 criteria and systematic diagnoses were not performed, which may lead to an incomplete mental assessment of this vulnerable population. Thirdly, the study sample included all patients in active treatment, end‐of‐life care and those in survivorship who came back to the hospital for additional treatment or recurrence of cancer. Therefore, the results of the study are not representative of a specific cancer group. It is important to have more well‐designed, multicenter studies with specific target groups to retest our findings in larger and more representative populations in Vietnam. The study only included Vietnamese cancer patients, so the cut‐off point of the DT has limited application to other cancer populations.

### 4.2 Clinical implications

Psychological distress is common among Vietnamese cancer patients but often underestimated and not detected by professionals due to lack of time and overload of clinical work. We found that the application of the DT followed by PL was simple and effective in Vietnamese patients. This result will constitute scientific evidence to advocate for the integration of psychological care into routine cancer care in Vietnam. After identifying patients’ unsatisfied needs, the cancer settings can provide appropriate psychosocial supports to enhance the quality of life. On that basis, there will be further intervention studies on psycho‐oncology for Vietnamese cancer patients to develop the comprehensive cancer care.

## 5 CONCLUSION

The study showed that the single‐item DT followed by PL with a cutoff of 4 is a simple and effective screening measure compared to previous longer measures used commonly in Vietnam to detect psychosocial distress in cancer patient. The PL identified patients with various problems contributing to psychological distress, including emotional problems, family issues, and practical and physical problems. The DT with cutoff score of 4 followed with PL is valid to screen psychological distress quickly and effectively among Vietnamese cancer patients.

## CONFLICTS OF INTEREST STATEMENT

The authors have no conflicts of interest.

## ETHICS STATEMENT

The study was approved by the Research Ethics Committee of the Vietnam National Cancer Hospital, Hanoi, Vietnam.

## Data Availability

The data used to support this study are included within the article.
